# Sex- and age-differences in supine positional obstructive sleep apnea in children and adults

**DOI:** 10.1007/s11325-025-03252-z

**Published:** 2025-02-17

**Authors:** Leping Li, Min Shi, David M. Umbach, Katelyn Bricker, Zheng Fan

**Affiliations:** 1 Biostatistics and Computational Biology Branch, National Institute of Environmental Health Sciences, Research Triangle Park, NC USA; 2 Division of Sleep Medicine, Chapel Hill, USA; 3 Department of Neurology, University of North Carolina at Chapel Hill, Chapel Hill, NC 27599 USA

**Keywords:** Age trajectory, Sex, Supine positional OSA, Exclusive supine positional OSA, Generalized linear mixed model

## Abstract

**Aim:**

To analyze sex differences in age trajectories of supine positional obstructive sleep apnea (POSA).

**Methods:**

We conducted retrospective analysis of polysomnography studies from 13,144 individuals aged from 2 to 103 years with at least 30 min of both supine and lateral sleep. We used generalized linear mixed-effects models to estimate position-specific mean apnea-hypopnea index (AHI) values and logistic regression to estimate the proportion with POSA or with exclusive POSA among individuals with obstructive sleep apnea (OSA). Predictors included sex, 5-y age group, sleep position, and their interactions.

**Results:**

Supine AHI was higher than lateral AHI regardless of age or sex except under age 5 y. The ratio of supine AHI to lateral AHI reliably exceeded 2 after age 30–35 in males and age 50–55 in females. For both sexes, the proportion with POSA among individuals with OSA increased rapidly with age until 30–35 and then stabilized. The proportion with POSA among individuals with OSA was significantly higher in males than females for each age group between 40 and 75 (*p* < 0.03). Among individuals with OSA in those 20 and older, the proportion with POSA was 64.6% (95% CI: 62.7%, 66.5%) in males and 55.8% (95% CI: 53.6%, 57.8%) in females. The proportion of individuals showing exclusive POSA also increased with age and peaked near 41% at age 15–20 in males and at age 20–25 in females.

**Conclusion:**

POSA becomes more common with age in both sexes; in women, its prevalence is generally lower but continues to increase after age 65.

**Supplementary Information:**

The online version contains supplementary material available at 10.1007/s11325-025-03252-z.

## Introduction

Sleep apnea is a common sleep disorder and is characterized by abnormalities of respiration during sleep. Sleep apnea causes normal breathing to stop or get very shallow. Apnea is a complete cessation of airflow that lasts at least 10 s and hypopnea is defined as a decrease in airflow of at least 30% and a decrease in oxygen saturation of at least 3% or an arousal, both of which last at least 10 s [1–5]. Obstructive sleep apnea (OSA), the most common form of sleep apnea, happens when the upper airway becomes blocked during sleep, thereby reducing or completely stopping airflow. The occurrence of OSA can depend on sleep position. The American Academy of Sleep Medicine (AASM) defines supine positional OSA (POSA) as a lower apnea/hypopnea index (AHI) in the non-supine position than in the supine position [6]. In practice, POSA is usually defined when, among those with OSA, AHI during sleep in the supine position is at least twice as high as AHI in other positions [7, 8].

POSA is thought to be caused by unfavorable airway geometry, reduced lung volume, inability of airway dilator muscles to adequately compensate for air loss in the supine position [9, 10]. POSA is prevalent [11–18]. In a cohort study of 574 OSA patients, 56% were found to have POSA [19]. A similar proportion (54%) was found in 6,437 OSA patients [13]. Moreover, about 20% of the adult OSA patients had exclusive supine positional OSA (e-POSA) (ratio of supine AHI to non-supine AHI ≥ 2 and non-supine AHI < 5) [13]. Heinzer et al. found that POSA was present in 53% of the general population and in 75% of OSA subjects and that the prevalence of e-POSA among OSA subjects was 36% [12].

POSA is treatable using a lateral positional device and/or a continuous positive airway pressure (CPAP) machine [14, 20–25]. Positional devices can be effective in selected patients and are more comfortable than CPAP machines. CPAP treatment alone may be unable to correct POSA in patients whose tongue remains prolapsed to block the airway. In fact, on supine sleep, the pressure from a CPAP machine when delivered with an orofacial interface may make the airway blockage even worse [26, 27]. For those patients, OSA is often thought to be best treated by CPAP with a nasal interface in combination with a lateral positional therapy device.

Various studies have shown that the prevalence of POSA and e-POSA among OSA patients is ~ 50–75% and 20–36%, respectively [11–16]. How the prevalence of these conditions changes with age is less known, however. To address those questions, we analyzed the relationships between sex and age and OSA-related parameters based on polysomnography (PSG) studies from 13,144 patients with at least 30 min of both supine and lateral sleep. We envision that our findings on the sex and age differences in POSA will augment the current knowledge on this topic and inform better clinical practice.

## Materials and methods

### In-laboratory PSG study

Each in-laboratory PSG study included an electroencephalogram with at least six channels (two frontal, two central, and two occipital), two electrooculograms, submental and bilateral tibialis surface electromyograms, and an electrocardiogram. Each PSG study also included airflow from nasal pressure and nasal/oral thermocouple, chest and abdominal movement via respiratory impedance plethysmography belts, end-tidal CO_2_ via a BCI capnograph sampled through a nasal cannula, and arterial blood oxygen via a finger oximetry. In selected patients, especially the very young, transcutaneous CO_2_ was used. Time-locked digital video was recorded with the PSG. The multi-channel polysomnogram was recorded digitally and stored using a Stellate Systems polygraph (Montreal, Quebec, Canada) from 2003 to 2012, a Grass Systems polygraph (model 7D) from 2013 to 2018, and a Natus polygraph (Natus^®^ SleepWorks™ PSG Software) after 2019.

The PSG study records four types of respiratory events – obstructive apnea, mixed apnea, central apnea, and hypopneas – from which AHI was calculated as the number of respiratory events per hour. Similarly, supine AHI and lateral AHI were calculated as the number of respiratory events per hour during supine and lateral sleep, respectively. Rapid eye movement (REM) AHI was calculated as the number of respiratory events per hour during REM sleep. All studies were manually scored using guidelines from the AASM scoring manuals current at the time of the PSG and interpreted by physicians who are board certified by the AASM (Supplementary text).

### Data, eligibility, and inclusion criteria

Our in-laboratory PSG studies include three types of studies: diagnostic, titration (treatment), and split night (diagnostic and treatment) studies. We included only diagnostic studies (*N* = 25,322) and the diagnostic portion of the split studies (*N* = 1,400), yielding 26,722 PSG studies. We excluded PSG studies that were missing information on sleep position (*N* = 1,623) or on BMI (body mass index) (*N* = 3,168). We excluded studies from patients under the age of 2 y (*N* = 1,091) and studies from patients with BMI values lower than 15 kg/m^2^ or greater than 100 kg/m^2^ (*N* = 705). We excluded PSG studies with fewer than 120 min of total sleep time (*N* = 445) and studies that did not have at least 30 min of sleep each in the supine and lateral positions (*N* = 2,143). Finally, we excluded studies when counts of positional respiratory events that exceeded the corresponding total event count (*N* = 75). These exclusions left 17,472 eligible PSG studies from 13,144 individual patients. For data analysis, we allowed only one PSG per patient by choosing the first PSG study by date for each patient. Thus, 13,144 patients contributed to data analysis for positional AHI. After applying those exclusion criteria and retaining only the first study for each patient, only full diagnostic PSG studies remained. For POSA analysis, we restricted our data to 8,568 of the 13,144 patients who were OSA positive.

For analysis of positional AHI during REM sleep, we required a PSG study to have at least 10 min of REM sleep in either the supine or lateral position. This additional restriction left 4,954 patients (699 with supine only, 1,256 with lateral only, and 2,999 with both positions) for positional REM AHI analyses.

### Definition of OSA and POSA

We denote participants under the age of 13 y as children and those 13 y or above as adults. For children, we used the following definitions for OSA. We defined OSA as present when AHI ≥ 1.5 and as absent otherwise. We defined POSA as AHI ≥ 1.5 with a ratio of supine AHI to lateral AHI ≥ 2, and we defined e-POSA as POSA positive and lateral AHI < 1.5. These definitions changed for adults. We defined OSA as present when AHI ≥ 5 and as absent otherwise. We defined POSA as overall AHI ≥ 5 and the ratio of supine AHI to lateral AHI ≥ 2, and we defined e-POSA as POSA positive and lateral AHI < 5.

### Statistical analysis

We used R (version 4.3.0) for all statistical analyses. We compared men and women using Wilcoxon’s rank sum test for continuous variables and Pearson’s chi-squared test for categorical variables. For regression analyses, we created 16 age categories: 2–5 y, 14 consecutive 5-year age groups starting at age 5 y, and 75 y or older.

To compare supine to lateral AHI, we used generalized linear mixed-effects models (GLMMs) for the negative binomial family using the logarithmic link function; these models took the total count of apnea and hypopnea events as the outcome variable with the logarithm of respective sleep time as the offset. Predictors included sex, age group, and sleep position, as well as their two-way and three-way interactions. For all models we also adjusted for year of study as a categorical variable to account for changes in technology over time. We included random intercepts for each individual in these models to account for the possible correlation between events in the supine and lateral positions for the same individuals.

We restricted POSA and e-POSA regression analyses to individuals with OSA and used logistic regression for analysis. The predictors included sex, age group and their interactions as well as year of study. Our primary analysis was descriptive and did not adjust for BMI; in secondary analyses, we adjusted for BMI by including BMI in the models though a 3-degree-of-freedom cubic spline.

Using output from the regression models, we constructed contrasts and confidence intervals to estimate the ratio of supine and lateral AHI and the proportion of POSA and e-POSA among PSGs with OSA for each sex and age-group combination. We applied the delta method [28] to estimate confidence limits for differences in proportions. To adjust for multiple testing when comparing males versus females across age groups, we employed the Benjamini-Hochberg procedure [29].

## Results

The final data set contained PSG studies from 13,144 individuals -- 6,967 women and 6,177 men. On average, women in the study were older and had higher BMI than the men (Table 1). Women in the study also slept longer on average and had lower average AHI than men. For numbers of individuals by sex and age group that contributed to different analyses, see Table S1a through Table S1e in Supplementary materials.

**Table 1:** Statistical summary of key variables from polysomnography used for data analysis

Characteristic	Female					Male					
	*N*	Mean	SD	Median	IQR	*N*	Mean	SD	Median	IQR	*P* value ^a^
Age (y)	6,967	41	21	44	30	6,177	38	23	40	41	< 0.001
BMI (kg/m^2^)	6,967	32	10	31	14	6,177	28	8	28	9	< 0.001
**Night total**											
Sleep time (min)	6,967	404	92	399	102	6,177	393	93	392	111	< 0.001
Event count	6,967	60	74	36	67	6,177	78	92	49	87	< 0.001
AHI (events/hr)	6,967	9	12	6	11	6,177	13	16	8	15	< 0.001
**Position-specific**											
Supine sleep time (min)	6,967	166	96	151	139	6,177	168	98	151	142	0.6
Supine events	6,967	33	46	17	40	6,177	47	59	28	56	< 0.001
Supine AHI	6,967	14	18	7	17	6,177	21	24	11	27	< 0.001
Lateral sleep time (min)	6,967	224	100	221	141	6,177	209	94	206	137	< 0.001
Lateral events	6,967	25	42	10	27	6,177	30	49	12	30	< 0.001
Lateral AHI	6,967	7	11	3	8	6,177	9	15	4	9	< 0.001
**Position- and REM-specific**											
Supine REM sleep time (min)	2,039	40	25	34	32	1,659	39	36	32	31	0.014
Supine REM events	2,039	15	19	9	19	1,659	14	18	8	17	0.13
Supine REM AHI	2,039	25	25	15	39	1,659	24	24	16	34	0.5
Lateral REM sleep time (min)	2,289	48	29	43	35	1,966	47	27	42	36	0.2
Lateral REM events	2,289	10	15	4	12	1,966	9	13	4	10	0.3
Lateral REM AHI	2,289	14	18	6	16	1,966	12	16	6	14	0.4
**OSA and subtypes** ^**b**^											
Positive for OSA ^c^	4,121	0.59	0.01	-	-	4,447	0.72	0	-	-	< 0.001
Positive for POSA ^d^	2,143	0.52	0.01	-	-	2,540	0.57	0	-	-	< 0.001
Positive for e-POSA ^d^	1,236	0.3	0.01	-	-	1,412	0.23	0	-	-	0.08

*N*, number of patients with an observed value for the variable; SD, standard deviation; IQR, interquartile range; BMI, body mass index; AHI, apnea-hypopnea index; REM, rapid eye movement; OSA, obstructive sleep apnea; POSA, supine positional OSA; e-POSA, exclusive supine positional OSA

^a^*P* values for comparisons of women to men from Wilcoxon’s rank sum test for continuous variables and from Pearson’s chi-squared test for categorical variables

^b^ For OSA and subtypes, the “Mean” column shows the estimated proportion and the “SD” column shows the standard error of the estimated proportion; the median and IQR are listed as ‘-‘ because the values are not useful for binary responses (presence or absence)

^c^ Denominator of the proportion is the number of patients: 6,967 for females; 6,177 for males

^d^ Denominator of the proportion is the number of patients positive for OSA: 4,121 for females; 4,447 for males

### Supine AHI and lateral AHI

**Fig. 1:**
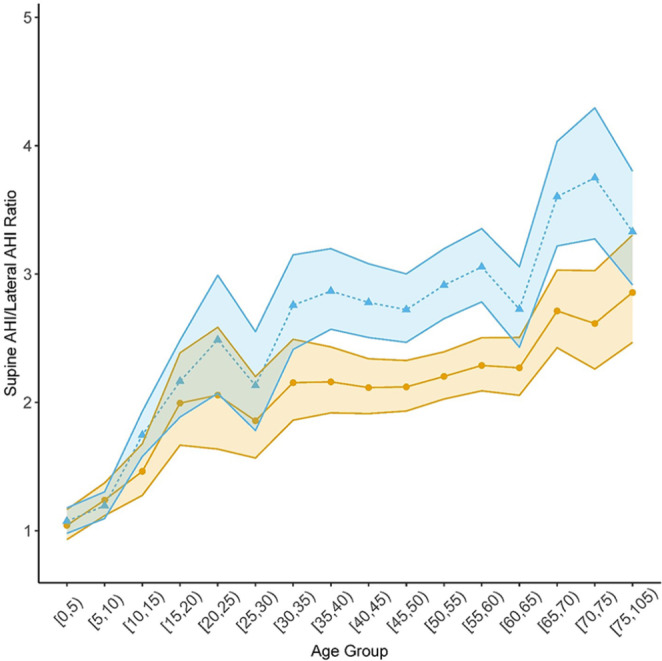
Estimates of the mean ratio of supine AHI to lateral AHI across age groups for males (blue triangles) and females (bronze circles) without any adjustment for BMI. These model-based estimates are from the negative binomial generalized linear mixed-effects model described in the Methods. All age groups span 5 y except the first (3 y) and last (30 y). Edges of the shaded areas connect pointwise 95% upper or lower confidence limits for each age group

In models without adjustment for BMI, estimates of mean supine AHI were higher than estimates of mean lateral AHI in both males and females across all age groups except 2–5 y as indicated by 95% confidence intervals for the respective supine-to-lateral ratios excluding 1 (Fig. 1; Table 2). The ratio of mean supine AHI to lateral AHI was reliably above 2 starting earlier in males (age group 30–35 y) than in females (age group 50–55 y). Males had significantly higher ratios of supine AHI to lateral AHI than females from age 30 y through 75 y. For those under age 20 y, the mean ratio of supine AHI to lateral AHI was estimated to be only 1.07 times higher in males than females (95% CI: 0.98, 1.16; *p* = 0.15); whereas, for those age 20 y and older, the mean ratio of supine AHI to lateral AHI was estimated to be 1.28 times higher in males than in females (95% CI: 1.21, 1.35; *p* < 0.0001).

**Table 2:** Estimates of the mean ratio of supine AHI to lateral AHI and of the ratio of the sex-specific ratios

Age Group	Male		Female		Ratio of mean ratio in M to that in F		
	Mean	95% CI	Mean	95% CI	Estimate	95% CI	*P* value ^a^
[2,5)	1.06	(0.96, 1.16)	1.06	(0.95, 1.19)	1.00	(0.86, 1.15)	0.96
[5,10)	1.17	(1.07, 1.28)	1.20	(1.07, 1.33)	0.98	(0.85, 1.13)	0.82
[10,15)	1.73	(1.55, 1.92)	1.48	(1.29, 1.71)	1.16	(0.97, 1.39)	0.15
[15,20)	2.22	(1.93, 2.56)	2.05	(1.71, 2.46)	1.08	(0.86, 1.36)	0.58
[20,25)	2.56	(2.11, 3.09)	2.06	(1.63, 2.62)	1.24	(0.91, 1.68)	0.23
[25,30)	2.12	(1.76, 2.54)	1.82	(1.52, 2.17)	1.17	(0.90, 1.50)	0.29
[30,35)	2.76	(2.41, 3.16)	2.15	(1.85, 2.50)	1.29	(1.05, 1.57)	0.03
[35,40)	2.87	(2.57, 3.21)	2.16	(1.91, 2.44)	1.33	(1.13, 1.57)	0.0017
[40,45)	2.81	(2.52, 3.12)	2.16	(1.94, 2.40)	1.30	(1.12, 1.51)	0.0015
[45,50)	2.71	(2.46, 3.00)	2.09	(1.90, 2.30)	1.30	(1.13, 1.49)	0.0007
[50,55)	2.88	(2.61, 3.17)	2.22	(2.04, 2.42)	1.29	(1.14, 1.47)	0.0007
[55,60)	3.03	(2.75, 3.34)	2.30	(2.09, 2.52)	1.32	(1.15, 1.51)	0.0007
[60,65)	2.70	(2.40, 3.05)	2.24	(2.02, 2.48)	1.21	(1.03, 1.42)	0.03
[65,70)	3.63	(3.23, 4.07)	2.65	(2.35, 2.98)	1.37	(1.16, 1.62)	0.0007
[70,75)	3.67	(3.19, 4.23)	2.46	(2.11, 2.88)	1.49	(1.21, 1.84)	0.0007
[75,105)	3.21	(2.80, 3.69)	2.73	(2.34, 3.19)	1.18	(0.96, 1.45)	0.18

These model-based estimates are from a negative binomial generalized linear mixed-effects model as described in the Methods without any adjustment for BMI (body mass index). All age groups span 5 y except the first (3 y) and last (30 y). AHI, apnea-hypopnea index. M, male; F, female

^a^*P* value adjusted for multiple testing using Benjamini-Hochberg procedure to control the false discovery rate

After adjustment for BMI, estimated age trajectories in the ratio of supine AHI to lateral AHI were similar in shape to those without BMI adjustment but with less pronounced differences between males and females (compare Fig. 1 with Fig. S1 in Supplementary materials and Table 2 with Table S2 in Supplementary materials).

The pattern of age- and sex-specific estimates of the ratio of supine REM AHI to lateral REM AHI mimicked the corresponding patterns of the ratio of supine AHI to lateral AHI both with and without adjusting for BMI (Fig. S2 and Fig. S3, respectively, in Supplementary materials).

### Proportion with POSA

**Fig. 2:**
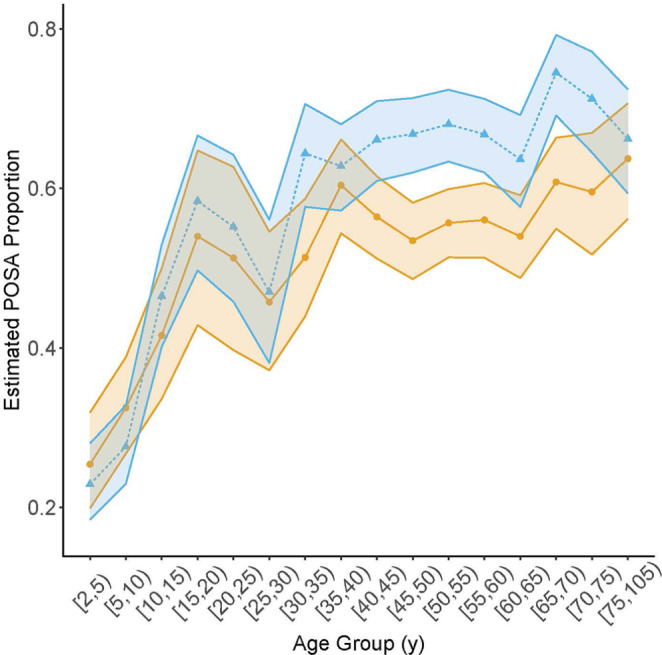
Estimates of the proportion of patients with POSA among those with OSA across age groups for males (blue triangles) and females (bronze circles) without any adjustment for BMI. These model-based estimates are from the logistic regression model described in the Methods. All age groups span 5 y except the first (3 y) and last (30 y). Edges of the shaded areas connect pointwise 95% upper or lower confidence limits for each age group

Analyses of POSA were limited to individuals with OSA -- 4,121 females and 4,447 males. In models without adjustment for BMI, the estimated proportion with POSA among individuals with OSA increased rapidly with age initially, until approximately 20–35 yrs old, and increased more slowly afterwards (Fig. 2; Table 3). Before the age of 30, the proportion with POSA among individuals with OSA were similar in both males and females; however, after the age of 40, the proportion with POSA was significantly higher in males than in females until age 75 (Table 3). Combining age groups below age 20 y, the proportion with POSA among individuals with OSA was similar in males (37.9%, 95% CI: 34.4%, 41.6%) and females (37.8%, 95% CI: 33.5%, 42.3%). Combining older age groups, the proportion of individuals with POSA in males was 64.6% (95% CI: 62.7%, 66.5%), significantly higher than that (55.8%, 95% CI: 53.6%, 57.8%) in females with a difference of 8.9% (95% CI: 6.2%, 11.6%; *p* < 0.0001). After the age of 65, the proportion of POSA showed an upward trend in females but a downward trend in males with both proportions similar in the oldest age group.

**Table 3:** Estimates of the proportion of patients with POSA among those with OSA and of the difference between the sex-specific proportions

Age Group	Male		Female		Difference in proportions between M and F		
	Mean	95% CI	Mean	95% CI	Estimate	95% CI	*P* value ^a^
[2,5)	0.23	(0.18, 0.28)	0.25	(0.20, 0.32)	-0.03	(-0.10, 0.05)	0.53
[5,10)	0.28	(0.23, 0.33)	0.32	(0.27, 0.39)	-0.05	(-0.12, 0.03)	0.53
[10,15)	0.46	(0.40, 0.53)	0.42	(0.34, 0.50)	0.05	(-0.05, 0.15)	0.53
[15,20)	0.58	(0.50, 0.67)	0.54	(0.43, 0.65)	0.04	(-0.09, 0.18)	0.53
[20,25)	0.55	(0.46, 0.64)	0.51	(0.40, 0.63)	0.04	(-0.11, 0.19)	0.67
[25,30)	0.47	(0.38, 0.56)	0.46	(0.37, 0.55)	0.01	(-0.11, 0.14)	0.85
[30,35)	0.64	(0.58, 0.71)	0.51	(0.44, 0.59)	0.13	( 0.03, 0.23)	0.02
[35,40)	0.63	(0.57, 0.68)	0.60	(0.54, 0.66)	0.02	(-0.06, 0.10)	0.67
[40,45)	0.66	(0.61, 0.71)	0.56	(0.51, 0.62)	0.10	( 0.03, 0.17)	0.02
[45,50)	0.67	(0.62, 0.71)	0.53	(0.49, 0.58)	0.13	( 0.07, 0.20)	0.001
[50,55)	0.68	(0.63, 0.72)	0.56	(0.51, 0.60)	0.12	( 0.06, 0.19)	0.001
[55,60)	0.67	(0.62, 0.71)	0.56	(0.51, 0.61)	0.11	( 0.04, 0.17)	0.004
[60,65)	0.64	(0.58, 0.69)	0.54	(0.49, 0.59)	0.10	( 0.02, 0.17)	0.03
[65,70)	0.75	(0.69, 0.79)	0.61	(0.55, 0.66)	0.14	( 0.06, 0.21)	0.002
[70,75)	0.71	(0.65, 0.77)	0.60	(0.52, 0.67)	0.12	( 0.02, 0.22)	0.03
[75,105)	0.66	(0.59, 0.72)	0.64	(0.56, 0.71)	0.02	(-0.07, 0.12)	0.67

These model-based estimates are from a logistic regression model as described in the Methods without any adjustment for BMI. All age groups span 5 y except the first (3 y) and last (30 y). POSA, supine positional obstructive sleep apnea; OSA, obstructive sleep apnea; M, male; F, female

^a^*P* value adjusted for multiple testing using Benjamini-Hochberg procedure to control the false discovery rate

After adjustment for BMI, estimated age trajectories in the proportion of individuals with POSA were similar in shape to those without BMI adjustment but differences in proportions between males and females were attenuated (compare Fig. 2 with Fig. S4 in Supplementary materials and Table 3 with Table S3 in Supplementary materials).

### Proportion with e-POSA

**Fig. 3:**
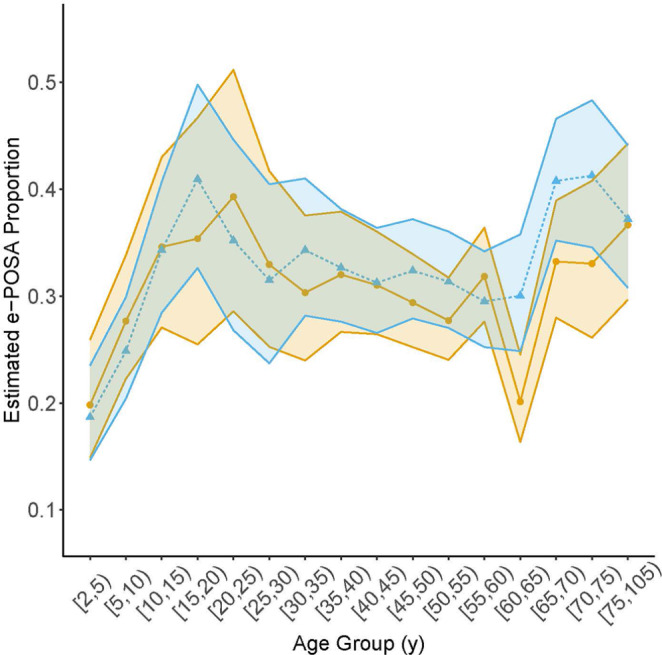
Estimates of the proportion of patients with e-POSA among those with OSA across age groups for males (blue triangles) and females (bronze circles) without any adjustment for BMI. These model-based estimates are from the logistic regression model described in the Methods. All age groups span 5 y except the first (3 y) and last (30 y). Edges of the shaded areas connect pointwise 95% upper or lower confidence limits for each age group

Analyses of e-POSA involved the same individuals as analyses of POSA. In models without adjustment for BMI, the estimated proportion with e-POSA among individuals with OSA were generally higher in males than in females for most age groups, although none of the differences was significant after adjusting for multiple testing (Table 4). The proportion with e-POSA increased with age in younger age groups and peaked in the 15–20 y age group for males at 41% and in the 20–25 y age group for females at 39% (Fig. 3; Table 4). The proportion with e-POSA was relatively stable, though with a slight downward trend, in both males and females between ages of 20 y and 65 y but increased after that (Fig. 3). Combining all age groups below 20 y, the overall mean proportion with e-POSA in males (28.9%, 95% CI: 25%, 33.1%) was nearly the same as that in females (28.9%, 95% CI: 25.7%, 32.4%), (*p* = 0.99 for sex difference). Combining all age groups older than age 20 y, the overall mean proportion with e-POSA in males (33.9%, 95% CI: 32.0%, 35.8%) was significantly higher than that in females (31.3%, 95% CI: 29.3%, 33.3%) with a difference of 2.6% (95% CI: 0.0%, 5.2%, *p* = 0.05).

**Table 4:** Estimates of the proportion of patients with e-POSA among those with OSA and of the difference between the sex-specific proportions

Age group	Male		Female		Difference in proportions between M and F		
	Mean	95% CI	Mean	95% CI	Estimate	95% CI	*P* value ^a^
[2,5)	0.19	(0.15, 0.24)	0.20	(0.15, 0.26)	-0.01	(-0.08, 0.06)	0.96
[5,10)	0.25	(0.20, 0.30)	0.28	(0.22, 0.34)	-0.03	(-0.10, 0.04)	0.88
[10,15)	0.34	(0.28, 0.41)	0.35	(0.27, 0.43)	0.00	(-0.10, 0.10)	0.96
[15,20)	0.41	(0.33, 0.50)	0.35	(0.25, 0.47)	0.06	(-0.08, 0.19)	0.88
[20,25)	0.35	(0.27, 0.45)	0.39	(0.29, 0.51)	-0.04	(-0.19, 0.10)	0.87
[25,30)	0.31	(0.24, 0.40)	0.33	(0.25, 0.42)	-0.01	(-0.13, 0.10)	0.95
[30,35)	0.34	(0.28, 0.41)	0.30	(0.24, 0.38)	0.04	(-0.05, 0.13)	0.79
[35,40)	0.33	(0.28, 0.38)	0.32	(0.27, 0.38)	0.01	(-0.07, 0.08)	0.95
[40,45)	0.31	(0.27, 0.36)	0.31	(0.26, 0.36)	0.00	(-0.07, 0.07)	0.95
[45,50)	0.32	(0.28, 0.37)	0.29	(0.25, 0.34)	0.03	(-0.03, 0.09)	0.79
[50,55)	0.31	(0.27, 0.36)	0.28	(0.24, 0.32)	0.04	(-0.02, 0.10)	0.67
[55,60)	0.3	(0.25, 0.34)	0.32	(0.28, 0.36)	-0.02	(-0.09, 0.04)	0.79
[60,65)	0.3	(0.25, 0.36)	0.20	(0.16, 0.25)	0.10	( 0.03, 0.17)	0.05
[65,70)	0.41	(0.35, 0.47)	0.33	(0.28, 0.39)	0.08	( 0.00, 0.15)	0.37
[70,75)	0.41	(0.35, 0.48)	0.33	(0.26, 0.41)	0.08	(-0.02, 0.18)	0.45
[75,105)	0.37	(0.31, 0.44)	0.37	(0.30, 0.44)	0.01	(-0.09, 0.10)	0.95

These model-based estimates are from a logistic regression model as described in the Methods without any adjustment for BMI. All age groups span 5 y except the first (3 y) and last (30 y). e-POSA, exclusive supine positional obstructive sleep apnea; OSA, obstructive sleep apnea; M, male; F, female

^a^*P* value adjusted for multiple testing using Benjamini-Hochberg procedure to control the false discovery rate

After adjustment for BMI, estimated age trajectories in the proportion with e-POSA among individuals with OSA showed the same general pattern as those without BMI adjustment and differences in proportions between males and females remained small across age groups (compare Fig. 3 with Fig. S5 in Supplementary materials and Table 4 with Table S4 in Supplementary materials).

## Discussion

Males are more susceptible to POSA than females [30, 31], however, the age trajectories of the sex differences in POSA are less clear. In this study, we estimated trajectories for AHI, POSA, and e-POSA using retrospective data from PSG studies on 13,144 individuals with at least 30 min of both supine and lateral sleep for AHI, POSA, and e-POSA analyses. We grouped the data into 5-y age bins and estimated the mean values of sex-specific POSA-related parameters in each age group.

We found that the estimated mean ratio of supine AHI to lateral AHI generally exceeded one across sex and age groups, indicating that on sleeping on one’s back is associated with higher AHI than sleeping on one’s side, consistent with prior findings [11–16, 19]. In children and young adults, the ratio of mean supine AHI to mean lateral AHI increased with age similarly for both sexes. Likewise, the proportion of POSA also increased with age in children and young adults (under 25) similarly for both sexes. On the other hand, age was less strongly related to POSA among adults than among children. For adults, both the ratio of supine AHI to lateral AHI and the proportion of POSA remained relatively unchanged with age between the age groups of 30–35 and 60–65.

On average, males had a higher ratio of supine AHI to lateral AHI than females, and males with OSA had higher proportion of POSA than women with OSA. After age 20 y, the average proportions of POSA among those with OSA were 65% and 56% in males and females, respectively. Our estimates for males were slightly higher than the overall 56% in 574 OSA patients [19] and the 54% in 6,437 patients with mild-to-severe OSA [13] but lower than the 75% estimated in a large population-based study of 1,719 subjects [12]. The differences may arise from heterogeneity among the populations sampled in these studies.

Our results also consistent with previous findings that OSA is more severe and POSA more common in men than women [19, 32, 33]. This observation has been attributed in part to the tendency of men to have airways that are larger but more collapsible during mandibular movement than women [33].

The proportion of e-POSA among those with OSA increased with age until about age 20 and then decreased slightly with age among both men and women until about age 65. The decrease may represent a transfer of e-POSA cases to POSA cases. If e-POSA represents a mild form of POSA, being present only during supine sleep, it may progress with age to a more severe form POSA characterized by increases in AHI during both supine and lateral sleep. This idea, however, does not explain with our observation that the proportion of e-POSA and of POSA both increased simultaneously in older women [34].

We did not attempt to probe the causal relationships between age and sex and POSA. Our primary analyses were descriptive as they did not adjust for the possible confounding influence of BMI on the relationships between AHI or OSA and age and sex. A previous report indicates that BMI is inversely correlated with the ratio of supine AHI to lateral AHI [19]. These relationships suggest that adjustment for BMI when modelling age and sex associations with OSA-related parameters may tend to attenuate male-to-female differences. In secondary analyses where we adjusted for BMI, this attenuation was evident.

### Strengths and limitations

Our study has important strengths. First, it is one of the largest studies of OSA; it includes PSG studies from 13,144 individuals, albeit with smaller numbers for supine- or lateral-specific variables. Second, our statistical analyses based on generalized linear models allowed us to estimate age- and sex-specific POSA-related parameters across the life course. We believe our study is the first to provide those estimates.

Our study also has several limitations. First, our data from a referral sleep clinic may have skewed our sample of patients toward the more severe spectrum. Second, our analysis included PSG studies spanning 17 years during which PSG technologies and scoring criteria changed. Although we tried to adjust for such technology changes in our statistical models, those adjustments are unlikely to fully account for changes in scoring criteria during the study period that may have influenced scoring of hypopnea. Third, we limited our analyses of POSA to those with at least 30 min of sleep in both supine and lateral positions. Longer and roughly equal amounts of time in both supine and lateral sleep would be preferred. Lastly, we did not adjust for potential confounders such as medical conditions, medication history, and demographic information such as race, education, smoking, and drinking.

## Conclusions

Our findings suggest that the prevalence POSA as well as the ratio of supine AHI to lateral AHI increase with age for both sexes. Nevertheless, men and women have somewhat different trajectories. Awareness of those age and sex differences should aid clinicians who treat patients of all ages. For example, in our data, the proportion of POSA in elderly women appears to continue to rise past age 65, whereas in men, the proportion showed a sign of declining after the age. Increased awareness by clinicians to this population of patients could improve patients’ care. Although CPAP remains the most effective treatment for OSA, combinational therapy including lateral positional therapy should also be considered to improve CPAP compliance and effectiveness, especially for the elderly who are presumed to be less likely to tolerate CPAP.

In summary, supine AHI was generally higher than lateral AHI for both sexes. The proportion with POSA among those with OSA increased rapidly with age until age 30–35 y and significantly higher in males than females among those 20 y or older. The proportion with e-POSA also increased with age among the young and peaked earlier in males (age 15–20 y) than in females (age 20–25 y). The prevalence of POSA was generally lower in females but its prevalence in elderly women continued to increase with age.

## Electronic supplementary material

Below is the link to the electronic supplementary material.

Supplementary Material 1

## Data Availability

The processed data used in the analysis can be requested from the corresponding author.

## References

[1] Panossian LA, Avidan AY (2009) Review of sleep disorders. Med Clin North Am 93:2:407– 25, ix. 10.1016/j.mcna.2008.09.001 10.1016/j.mcna.2008.09.001 19272516

[2] Duran J, Esnaola S, Rubio R, Iztueta A (2001) Obstructive sleep apnea-hypopnea and related clinical features in a population-based sample of subjects aged 30 to 70 year. Am J Respir Crit Care Med 163(3 Pt 1):685–689. 10.1164/ajrccm.163.3.2005065 11254524 10.1164/ajrccm.163.3.2005065

[3] Franklin KA, Lindberg E (2015) Obstructive sleep apnea is a common disorder in the population-a review on the epidemiology of sleep apnea. J Thorac Dis 7(8):1311–1322. 10.3978/j.issn.2072-1439.2015.06.11 10.3978/j.issn.2072-1439.2015.06.11 PMC4561280 26380759

[4] Tishler PV, Larkin EK, Schluchter MD, Redline S (2003) Incidence of sleep-disordered breathing in an urban adult population: the relative importance of risk factors in the development of sleep-disordered breathing. JAMA 289:17:2230–2237. 10.1001/jama.289.17.2230 12734134 10.1001/jama.289.17.2230

[5] Levy P, Kohler M, McNicholas WT, Barbe F, McEvoy RD, Somers VK et al (2015) Obstructive sleep apnoea syndrome. Nat Rev Dis Primers 1:15015. 10.1038/nrdp.2015.15 27188535 10.1038/nrdp.2015.15

[6] Sleep-related breathing (1999) Disorders in adults: recommendations for syndrome definition and measurement techniques in clinical research. The report of an American Academy of Sleep Medicine Task Force. Sleep 22:5667–5689 10450601

[7] Cartwright RD (1984) Effect of sleep position on sleep apnea severity. Sleep 7:2. 10.1093/sleep/7.2.110 10.1093/sleep/7.2.110 6740055

[8] Lloyd SR, Cartwright RD (1987) Physiologic basis of therapy for sleep apnea. Am Rev Respir Dis 136(2):525–526. 10.1164/ajrccm/136.2.525b 10.1164/ajrccm/136.2.525b 3619217

[9] Joosten SA, O’Driscoll DM, Berger PJ, Hamilton GS (2014) Supine position related obstructive sleep apnea in adults: pathogenesis and treatment. Sleep Med Rev 18:1:7–17. 10.1016/j.smrv.2013.01.005 23669094 10.1016/j.smrv.2013.01.005

[10] Landry SA, Beatty C, Thomson LDJ, Wong AM, Edwards BA, Hamilton GS et al (2023) A review of supine position related obstructive sleep apnea: classification, epidemiology, pathogenesis and treatment. Sleep Med Rev 72:101847. 10.1016/j.smrv.2023.101847 37722317 10.1016/j.smrv.2023.101847

[11] Mador MJ, Kufel TJ, Magalang UJ, Rajesh SK, Watwe V, Grant BJ (2005) Prevalence of positional sleep apnea in patients undergoing polysomnography. Chest 128:4:2130–2137. 10.1378/chest.128.4.2130 16236865 10.1378/chest.128.4.2130

[12] Heinzer R, Petitpierre NJ, Marti-Soler H, Haba-Rubio J (2018) Prevalence and characteristics of positional sleep apnea in the HypnoLaus population-based cohort. Sleep Med 48:157–162. 10.1016/j.sleep.2018.02.011 10.1016/j.sleep.2018.02.011 29957486

[13] Sabil A, Blanchard M, Trzepizur W, Goupil F, Meslier N, Paris A et al (2020) Positional obstructive sleep apnea within a large multicenter French cohort: prevalence, characteristics, and treatment outcomes. J Clin Sleep Med 16:122037–122046. 10.5664/jcsm.8752 10.5664/jcsm.8752 PMC7848932 32804071

[14] Permut I, Diaz-Abad M, Chatila W, Crocetti J, Gaughan JP, D’Alonzo GE et al (2010) Comparison of positional therapy to CPAP in patients with positional obstructive sleep apnea. J Clin Sleep Med 6:3238–3243 PMC2883034 20572416

[15] Laub RR, Mikkelsen KL, Tønnesen P (2015) Prevalence of positional obstructive sleep apnea and patients characteristics using various definitions. Eur Respir J 46(suppl 59):PA2372. 10.1183/13993003.congress-2015.PA2372

[16] Wali SO, AlQassas I, Qanash S, Mufti H, Alamoudi M, Alnowaiser M et al (2023) The prevalence of positional obstructive sleep apnoea in a sample of the Saudi Population. J Epidemiol Glob Health 13(1):129–139. 10.1007/s44197-023-00089-1 10.1007/s44197-023-00089-1 PMC10006370 36705890

[17] Richard W, Kox D, den Herder C, Laman M, van Tinteren H, de Vries N (2006) The role of sleep position in obstructive sleep apnea syndrome. Eur Arch Otorhinolaryngol 263(10):946–950. 10.1007/s00405-006-0090-2 10.1007/s00405-006-0090-2 16802139

[18] Pevernagie DA, Shepard JW Jr (1992) Relations between sleep stage, posture and effective nasal CPAP levels in OSA. Sleep 15(2):162–167. 10.1093/sleep/15.2.162 10.1093/sleep/15.2.162 1579791

[19] Oksenberg A, Silverberg DS, Arons E, Radwan H (1997) Positional vs nonpositional obstructive sleep apnea patients: anthropomorphic, nocturnal polysomnographic, and multiple sleep latency test data. Chest 112(3):629–639. 10.1378/chest.112.3.629 10.1378/chest.112.3.629 9315794

[20] de Vries GE, Hoekema A, Doff MH, Kerstjens HA, Meijer PM, van der Hoeven JH et al (2015) Usage of positional therapy in adults with obstructive sleep apnea. J Clin Sleep Med 11:2:131–137. 10.5664/jcsm.4458 25406271 10.5664/jcsm.4458 PMC4298770

[21] Troester N, Palfner M, Dominco M, Wohlkoenig C, Schmidberger E, Trinker M et al (2017) Positional therapy in sleep apnoea - one fits all? What determines success in positional therapy in sleep apnoea syndrome. PLoS ONE 12:4e0174468. 10.1371/journal.pone.0174468 10.1371/journal.pone.0174468 PMC5390972 28406975

[22] Vecchierini MF, Attali V, Collet JM, d’Ortho MP, Goutorbe F, Kerbrat JB et al (2019) Sex differences in mandibular repositioning device therapy effectiveness in patients with obstructive sleep apnea syndrome. Sleep Breath 23(3):837–848. 10.1007/s11325-018-1766-8 10.1007/s11325-018-1766-8 PMC6700234 30580418

[23] Dieltjens M, Vroegop AV, Verbruggen AE, Wouters K, Willemen M, De Backer WA et al (2015) A promising concept of combination therapy for positional obstructive sleep apnea. Sleep Breath 19(2):637–644. 10.1007/s11325-014-1068-8 10.1007/s11325-014-1068-8 PMC4873543 25335642

[24] Eijsvogel MM, Ubbink R, Dekker J, Oppersma E, de Jongh FH, van der Palen J et al (2015) Sleep position trainer versus tennis ball technique in positional obstructive sleep apnea syndrome. J Clin Sleep Med 11:2139–2147. 10.5664/jcsm.4460 10.5664/jcsm.4460 PMC4298771 25515276

[25] Ravesloot MJL, White D, Heinzer R, Oksenberg A, Pépin JL (2017) Efficacy of the New Generation of devices for positional therapy for patients with positional obstructive sleep apnea: a systematic review of the literature and Meta-analysis. J Clin Sleep Med 13:6813–6824. 10.5664/jcsm.6622 10.5664/jcsm.6622 PMC5443742 28212691

[26] Htun ZM, McCullough L, Lastra AC, Ineffective CPAP, Treatment After Effective CPAP, Titration (2020) Chest 158(6):e311-e5. 10.1016/j.chest.2020.07.030 10.1016/j.chest.2020.07.030 33280775

[27] Genta PR, Kaminska M, Edwards BA, Ebben MR, Krieger AC, Tamisier R et al (2020) The importance of Mask selection on continuous positive Airway pressure outcomes for obstructive sleep apnea. An official American thoracic Society Workshop Report. Ann Am Thorac Soc 17:10. 10.1513/AnnalsATS.202007-864ST 10.1513/AnnalsATS.202007-864ST PMC7640631 33000960

[28] Bishop YMMFS, Holland PW (1975) Discrete multivariate analysis: theory and practice. MIT, MIT

[29] Benjamini Y, Hochberg Y (1995) Controlling the false Discovery rate: a practical and powerful Approach to multiple testing. J Roy Stat Soc: Ser B (Methodol) 57:1:289–300. 10.1111/j.2517-6161.1995.tb02031.x

[30] Kapsimalis F, Kryger MH (2002) Gender and obstructive sleep apnea syndrome, part 1: clinical features. Sleep 25:4 12071542

[31] Lin CM, Davidson TM, Ancoli-Israel S (2008) Gender differences in obstructive sleep apnea and treatment implications. Sleep Med Rev 12(6):481–496. 10.1016/j.smrv.2007.11.003 10.1016/j.smrv.2007.11.003 PMC2642982 18951050

[32] O’Connor C, Thornley KS, Hanly PJ (2000) Gender differences in the polysomnographic features of obstructive sleep apnea. Am J Respir Crit Care Med 161:5:1465–1472. 10.1164/ajrccm.161.5.9904121 10806140 10.1164/ajrccm.161.5.9904121

[33] Mohsenin V (2003) Effects of gender on upper airway collapsibility and severity of obstructive sleep apnea. Sleep Med 4:6:523–529. 10.1016/s1389-9457(03)00168-0 14607346 10.1016/s1389-9457(03)00168-0

[34] Oksenberg A, Silverberg D, Offenbach D, Arons E (2006) Positional therapy for obstructive sleep apnea patients: a 6-month follow-up study. Laryngoscope 116:11:1995–2000. 10.1097/01.mlg.0000237674.66716.a7 10.1097/01.mlg.0000237674.66716.a7 17075418

