# Supplementary material for: Sex- and age-differences in supine positional obstructive sleep apnea in children and adults
Source: Sleep Breath. 2025 Feb 17;29(1):106. doi: 10.1007/s11325-025-03252-z (PMC11832621; doi:10.1007/s11325-025-03252-z)
Supplement: Supplementary file 1 — Supplementary Material 1 [file 11325_2025_3252_MOESM1_ESM.docx]

**Supplementary materials for**

**Sex- and Age-differences in Supine Positional Obstructive Sleep Apneas in Children and Adults**

Leping Li, Min Shi, David M. Umbach, Katelyn Bricker, Zheng Fan

**Supplementary Methods**

**Study protocol**

We conducted a retrospective review of in-laboratory sleep PSG data, approved by the Institutional Review Board of the University of North Carolina at Chapel Hill (UNC-CH) (IRB #21-1984) using studies carried out between January 2003 and July 2023 in an AASM accredited sleep laboratory at UNC-CH. The UNC-CH sleep lab serves patients from the entire state, both metropolitan and rural areas. Most patients were referred for sleep apnea evaluation.

**Scoring**

PSG studies before 2007 were scored using the guidelines from the Sleep Disorders Atlas Task Force of the American Sleep Disorders Association [1]. Studies from September 2007 until June 2016 were scored with the 2007 guidelines from the AASM scoring manuals [2]. Subsequent studies were scored with the 2016 guidelines [3]. The studies were manually scored using guidelines from the AASM scoring manuals current at the time of the PSG and interpreted by physicians who are board certified by the AASM.

**Supplementary Results**

**Supine REM AHI and lateral REM AHI**

The pattern of age- and sex-specific estimates of the ratio of supine REM AHI to lateral REM AHI mimicked the corresponding pattern of the ratio of supine AHI to lateral AHI when using models that did not adjust for BMI (compare **Fig.** **1** and **Fig. S2 in Supplementary materials**). Without BMI adjustment, supine REM AHI was higher than that of lateral REM AHI in both males and females across all age groups except 2-5 y for females and 5-10 y for males as indicated by 95% confidence limits for ratio of mean supine AHI to lateral AHI excluding 1 (**Table S5a**). The estimated ratio of supine REM AHI to lateral REM AHI increased with age in both sexes. Although the estimated ratio reached above 2 earlier in males (age group 15-20) than in females (age group 30-35), the ratio was reliably above 2, based on the confidence interval excluding 2, only about 20 y later, starting in age group 40-45 in males and in age group 50-55 in females. Males generally had higher ratios of supine REM AHI to lateral REM AHI than females across all age groups although the sex differences were statistically significant only for the 65-70 age group (**Table** **S5a**). Combining age groups below 20 y, the mean ratio of supine REM AHI to lateral REM AHI was 1.08 times higher in males than in females (95% CI: 0.95, 1.22, *P* = 0.23). For age groups 20 y and older, the mean ratio of supine REM AHI to lateral REM AHI was 1.29 times higher in males than in females (95% CI: 1.16, 1.43, *p* < 0.0001), an estimate near the corresponding estimate for the ratio of supine AHI to lateral AHI over all sleep stages.

Estimates of the ratio of supine REM AHI to lateral REM AHI based on models that adjusted for BMI showed broadly similar patterns to those based on models without BMI adjustment although the adjustment eliminated statistically significant differences between males and females (compare **Fig. S2** with **Fig. S3** and **Table S5a** with **Table S5b**).

**Supplementary Figures**

**
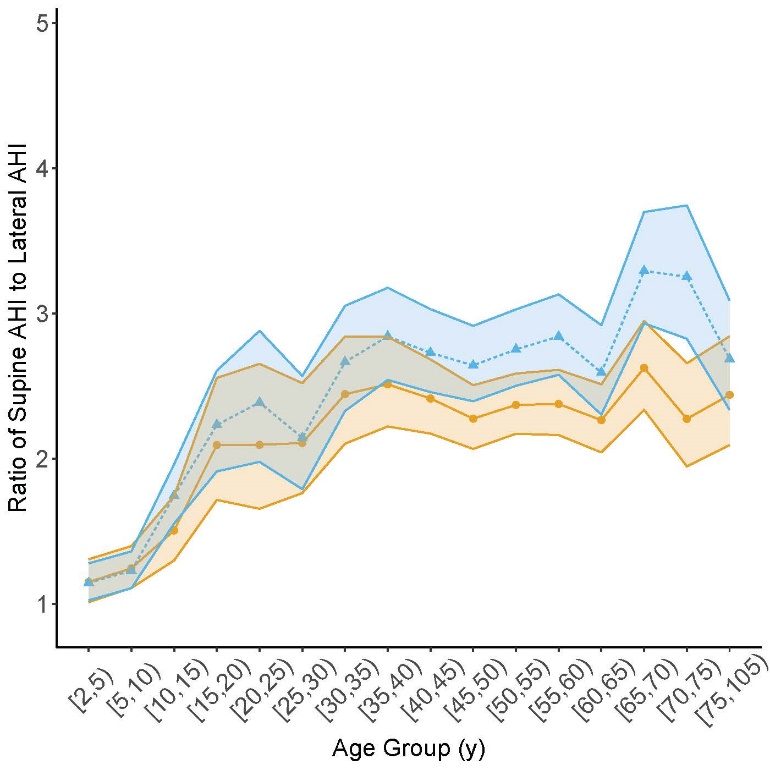
**

**Fig. S1.** Estimated mean ratio of supine AHI to lateral AHI in each age group in males (blue triangles) and females (bronze circles) after adjusting for the effect of BMI. These estimates are from the negative binomial generalized linear mixed-effects model described in the Methods. All age groups span 5 y except the first (3 y) and last (30 y). Edges of the shaded areas connect pointwise 95% upper or lower confidence limits for each age group.


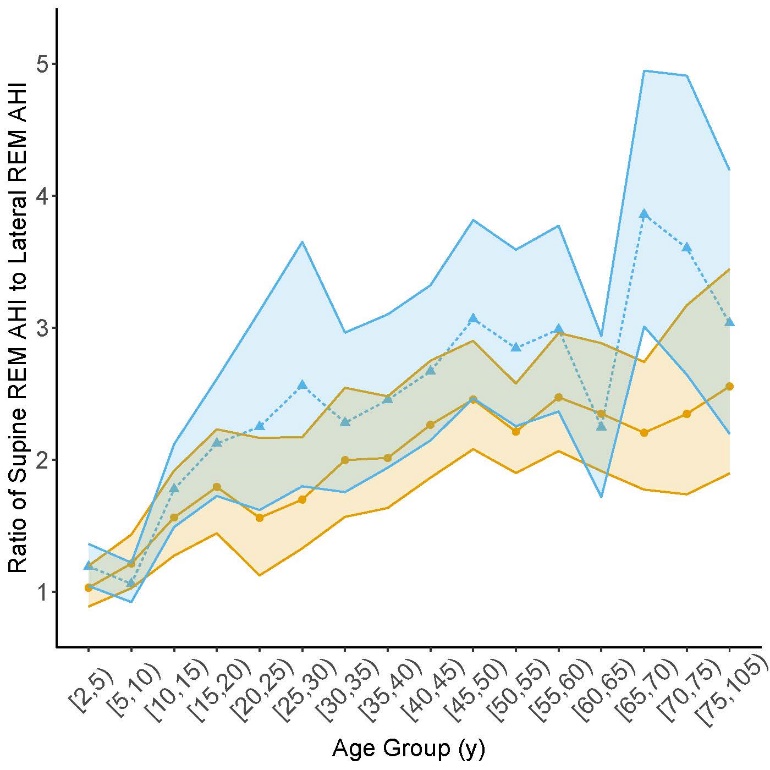


**Fig. S2**. Estimated mean ratio of supine REM AHI to lateral REM AHI in each age group in males (blue triangles) and females (bronze circles) without adjusting for the effect of BMI. These estimates are from the negative binomial generalized linear mixed-effects model described in the Methods. All age groups span 5 y except the first (3 y) and last (30 y). Edges of the shaded areas connect pointwise 95% upper or lower confidence limits for each age group.


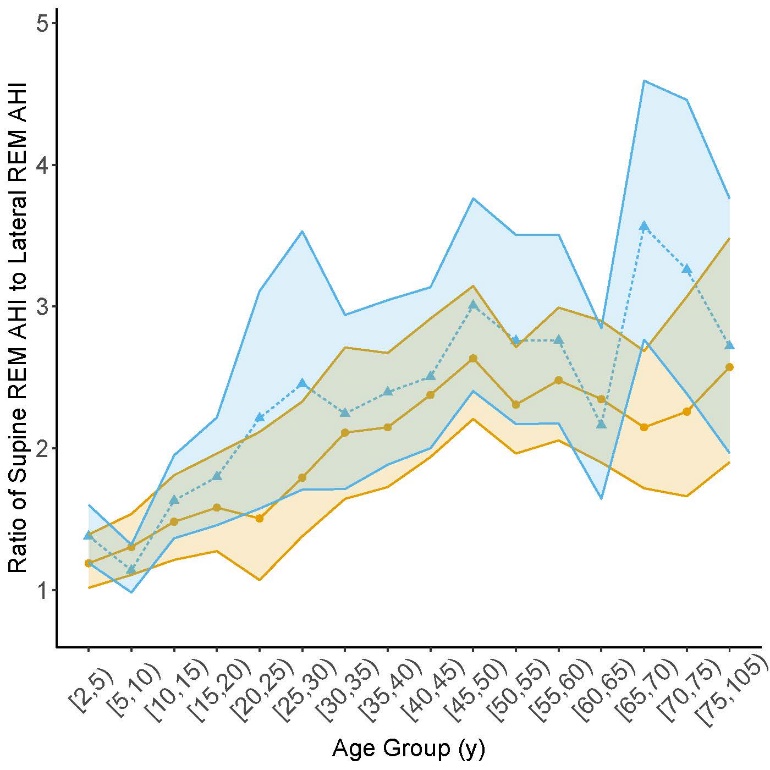


**Fig. S3**. Estimated mean ratio of supine REM AHI to lateral REM AHI in males (blue triangles) and females (bronze circles) in each age group after adjusting for the effect of BMI. These estimates are from the negative binomial generalized linear mixed-effects model described in the Methods. All age groups span 5 y except the first (3 y) and last (30 y). Edges of the shaded areas connect pointwise 95% upper or lower confidence limits for each age group.

**
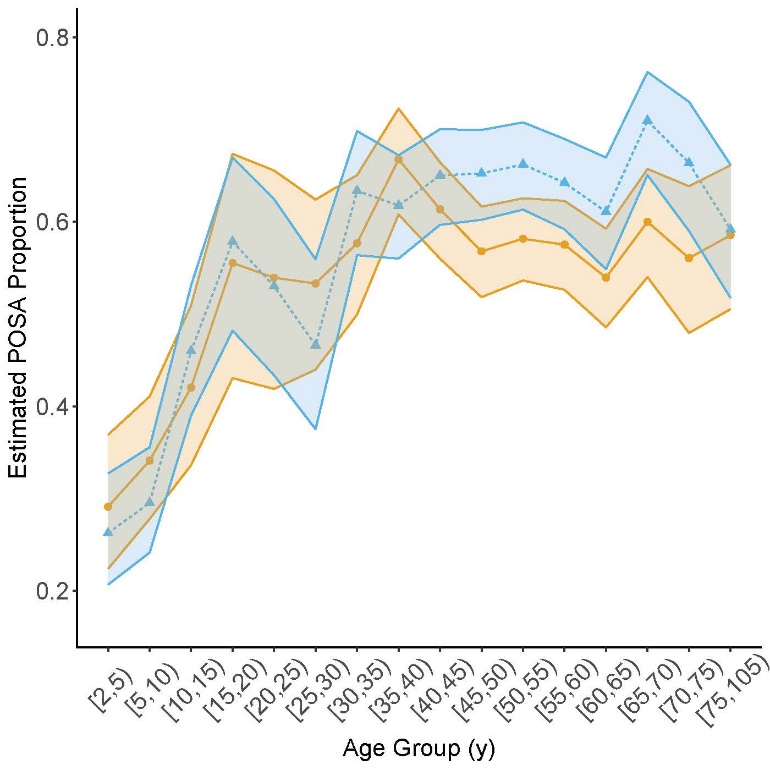
**

**Fig. S4.** Estimates of the proportion of patients with POSA among those with OSA across age groups in males (blue triangles) and females (bronze circles) after adjusting for the effect of BMI. These estimates are from logistic regression. All age groups span 5 y except the first (3 y) and last (30 y). Edges of the shaded areas connect the pointwise 95% upper or lower confidence limits for each age group.


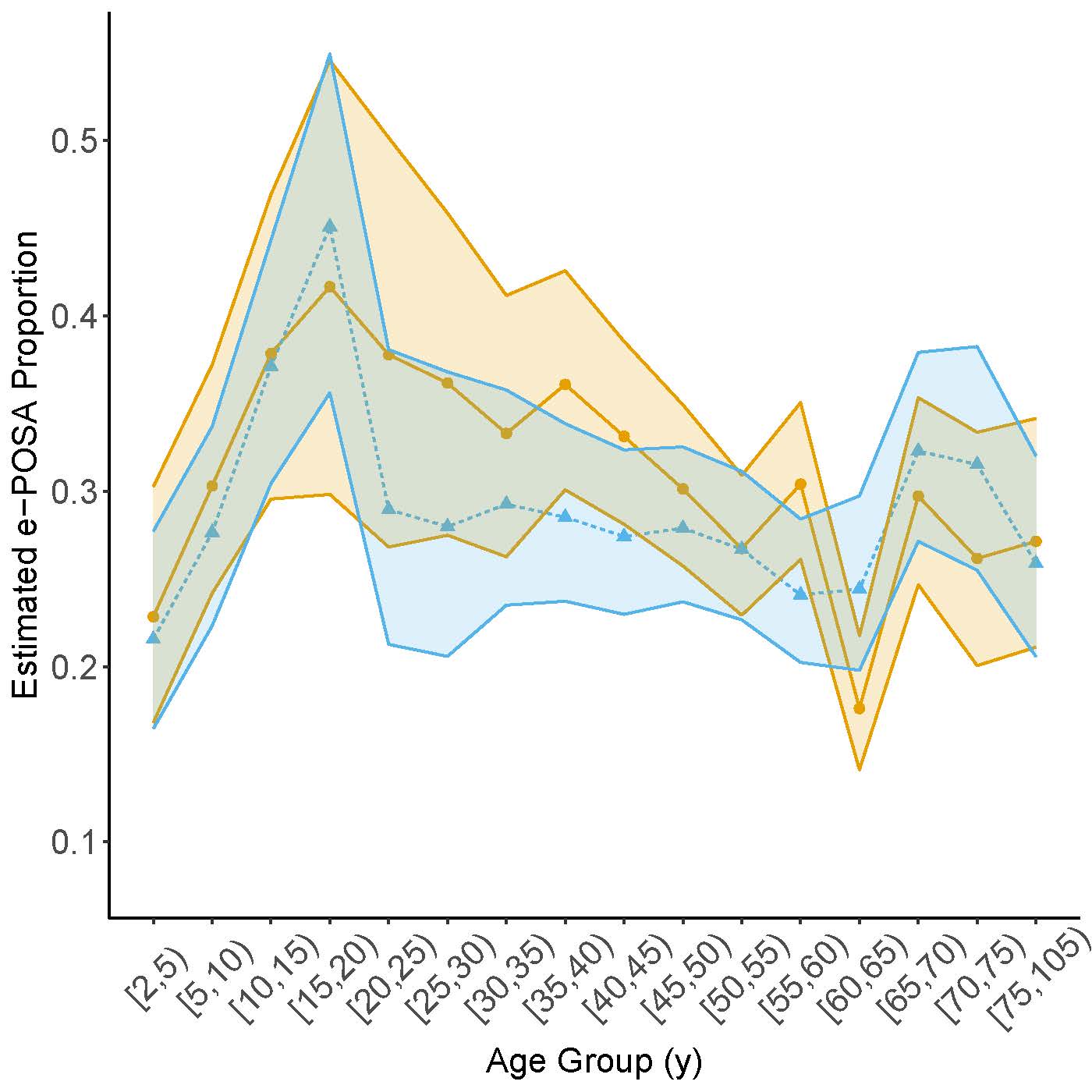


**Fig. S5.** Estimates of the proportion of patients with e-POSA among those with OSA across age groups in males (blue triangles) and females (bronze circles) after adjusting for the effect of BMI. These estimates are from logistic regression. All age groups span 5 y except the first (3 y) and last (30 y). Edges of the shaded areas connect the pointwise 95% upper or lower confidence limits for each age group.

**Supplementary Tables**

**Table S1**. Age group distribution. Left parenthesis indicates non-inclusion whereas square bracket indicates inclusion.

| **Age Group** | **F**, N = 10,404*^1^* | **M**, N = 9,033*^1^* |
| --- | --- | --- |
| [2,5) | 449 (4.3%) | 645 (7.1%) |
| [5,10) | 641 (6.2%) | 884 (9.8%) |
| [10,15) | 511 (4.9%) | 672 (7.4%) |
| [15,20) | 448 (4.3%) | 438 (4.8%) |
| [20,25) | 324 (3.1%) | 344 (3.8%) |
| [25,30) | 457 (4.4%) | 313 (3.5%) |
| [30,35) | 589 (5.7%) | 404 (4.5%) |
| [35,40) | 749 (7.2%) | 559 (6.2%) |
| [40,45) | 880 (8.5%) | 607 (6.7%) |
| [45,50) | 1,055 (10%) | 662 (7.3%) |
| [50,55) | 1,077 (10%) | 706 (7.8%) |
| [55,60) | 956 (9.2%) | 755 (8.4%) |
| [60,65) | 753 (7.2%) | 548 (6.1%) |
| [65,70) | 652 (6.3%) | 605 (6.7%) |
| [70,75) | 423 (4.1%) | 427 (4.7%) |
| [75,105) | 440 (4.2%) | 464 (5.1%) |

**Table S2**. Estimated ratio of supine REM AHI to lateral REM AHI ratio in each age group for males and females without adjusting for the effect of BMI.

| Age group | Mean supine-REM-AHI/lateral-REM-AHI | | M/F ratio (CI) | *P*^‡^ | Adj. *P*^†^ |
| --- | --- | --- | --- | --- | --- |
|  | Male | Female |  |  |  |
| [2,5) | 1.14 (0.99, 1.32) | 1.04 (0.89, 1.22) | 1.09 (0.88, 1.36) | 0.41 | 0.60 |
| [5,10) | 1.18 (1.03, 1.36) | 1.23 (1.04, 1.45) | 0.96 (0.78, 1.19) | 0.71 | 0.76 |
| [10,15) | 1.85 (1.55, 2.19) | 1.56 (1.28, 1.90) | 1.18 (0.91, 1.53) | 0.21 | 0.42 |
| [15,20) | 2.08 (1.68, 2.58) | 1.74 (1.40, 2.17) | 1.19 (0.88, 1.63) | 0.26 | 0.46 |
| [20,25) | 2.28 (1.66, 3.13) | 1.68 (1.25, 2.26) | 1.36 (0.88, 2.09) | 0.17 | 0.38 |
| [25,30) | 2.20 (1.57, 3.09) | 1.63 (1.29, 2.06) | 1.35 (0.90, 2.04) | 0.15 | 0.38 |
| [30,35) | 2.26 (1.75, 2.93) | 2.06 (1.64, 2.59) | 1.10 (0.78, 1.55) | 0.59 | 0.73 |
| [35,40) | 2.47 (1.97, 3.08) | 1.91 (1.57, 2.31) | 1.29 (0.97, 1.73) | 0.09 | 0.36 |
| [40,45) | 2.43 (1.97, 2.99) | 2.20 (1.85, 2.63) | 1.10 (0.84, 1.45) | 0.49 | 0.66 |
| [45,50) | 2.7 (2.18, 3.32) | 2.37 (2.02, 2.78) | 1.14 (0.87, 1.48) | 0.34 | 0.54 |
| [50,55) | 2.66 (2.15, 3.31) | 2.17 (1.87, 2.51) | 1.23 (0.95, 1.60) | 0.12 | 0.38 |
| [55,60) | 2.77 (2.25, 3.41) | 2.17 (1.84, 2.55) | 1.28 (0.98, 1.67) | 0.07 | 0.36 |
| [60,65) | 2.35 (1.83, 3.00) | 2.27 (1.88, 2.75) | 1.03 (0.76, 1.41) | 0.85 | 0.85 |
| [65,70) | 3.40 (2.72, 4.25) | 2.23 (1.84, 2.71) | 1.52 (1.13, 2.05) | 0.01 | 0.09 |
| [70,75) | 3.49 (2.65, 4.60) | 2.54 (1.98, 3.25) | 1.38 (0.95, 1.99) | 0.09 | 0.36 |
| [75,105) | 2.94 (2.23, 3.87) | 2.72 (2.10, 3.51) | 1.08 (0.74, 1.58) | 0.69 | 0.76 |

^‡^Generalized linear models; ^†^Benjamini-Hochberg Procedure

**Table S3**. Mean male to female ratio of the supine AHI to lateral AHI ratio and the supine REM AHI to lateral REM AHI ratio after adjusting for the effect of BMI.

|  | Supine AHI/lateral AHI | | | Supine REM AHI/lateral REM AHI | | |
| --- | --- | --- | --- | --- | --- | --- |
| Age group | M/F ratio (CI) | *P* | Adj. *P* | M/F ratio (CI) | *P‡* | Adj. *P†* |
| [2,5) | 1.03 (0.89, 1.19) | 0.70 | 0.74 | 1.08 (0.88, 1.34) | 0.46 | 0.73 |
| [5,10) | 0.97 (0.85, 1.11) | 0.63 | 0.71 | 0.97 (0.78, 1.19) | 0.75 | 0.89 |
| [10,15) | 1.19 (1.01, 1.41) | 0.04 | 0.11 | 1.13 (0.87, 1.47) | 0.35 | 0.71 |
| [15,20) | 1.08 (0.86, 1.35) | 0.52 | 0.63 | 1.15 (0.85, 1.56) | 0.37 | 0.71 |
| [20,25) | 1.10 (0.83, 1.47) | 0.50 | 0.63 | 1.44 (0.93, 2.23) | 0.10 | 0.64 |
| [25,30) | 1.00 (0.78, 1.27) | 0.98 | 0.98 | 1.19 (0.79, 1.81) | 0.40 | 0.71 |
| [30,35) | 1.08 (0.89, 1.31) | 0.438 | 0.63 | 0.96 (0.68, 1.37) | 0.84 | 0.89 |
| [35,40) | 1.13 (0.96, 1.33) | 0.133 | 0.24 | 1.16 (0.86, 1.57) | 0.32 | 0.71 |
| [40,45) | 1.12 (0.97, 1.29) | 0.120 | 0.24 | 0.93 (0.70, 1.22) | 0.59 | 0.85 |
| [45,50) | 1.15 (1.01, 1.31) | 0.039 | 0.11 | 1.03 (0.79, 1.35) | 0.82 | 0.89 |
| [50,55) | 1.18 (1.04, 1.34) | 0.008 | 0.045 | 1.17 (0.90, 1.52) | 0.24 | 0.71 |
| [55,60) | 1.21 (1.07, 1.38) | 0.0032 | 0.026 | 1.21 (0.93, 1.58) | 0.16 | 0.64 |
| [60,65) | 1.13 (0.98, 1.31) | 0.101 | 0.23 | 1.00 (0.73, 1.37) | 0.99 | 0.99 |
| [65,70) | 1.22 (1.04, 1.42) | 0.014 | 0.056 | 1.45 (1.08, 1.95) | 0.01 | 0.20 |
| [70,75) | 1.36 (1.12, 1.65) | 0.002 | 0.026 | 1.32 (0.92, 1.90) | 0.14 | 0.64 |
| [75,105) | 1.09 (0.90, 1.32) | 0.38 | 0.60 | 0.92 (0.63, 1.32) | 0.64 | 0.85 |

*‡*Generalized linear models; †Benjamini-Hochberg Procedure

**Table S4**. Estimated POSA proportions in males and females in each age group after adjusting for the effect of BMI.

| Age group | Female | Male | *P‡* | Adj. *P†* |
| --- | --- | --- | --- | --- |
| [2,5) | 0.26 (0.20, 0.33) | 0.26 (0.21, 0.32) | 0.898 | 0.90 |
| [5,10) | 0.35 (0.29, 0.42) | 0.29 (0.24, 0.35) | 0.104 | 0.41 |
| [10,15) | 0.40 (0.32, 0.48) | 0.46 (0.40, 0.53) | 0.205 | 0.41 |
| [15,20) | 0.54 (0.42, 0.65) | 0.59 (0.50, 0.68) | 0.407 | 0.54 |
| [20,25) | 0.54 (0.42, 0.65) | 0.52 (0.43, 0.62) | 0.830 | 0.91 |
| [25,30) | 0.54 (0.45, 0.63) | 0.48 (0.39, 0.57) | 0.337 | 0.51 |
| [30,35) | 0.59 (0.51, 0.66) | 0.63 (0.56, 0.69) | 0.444 | 0.53 |
| [35,40) | 0.67 (0.61, 0.72) | 0.62 (0.56, 0.67) | 0.229 | 0.39 |
| [40,45) | 0.61 (0.56, 0.66) | 0.64 (0.59, 0.69) | 0.436 | 0.53 |
| [45,50) | 0.58 (0.53, 0.62) | 0.65 (0.60, 0.70) | 0.024 | 0.07 |
| [50,55) | 0.58 (0.54, 0.63) | 0.66 (0.62, 0.71) | 0.013 | 0.07 |
| [55,60) | 0.57 (0.53, 0.62) | 0.65 (0.60, 0.70) | 0.025 | 0.07 |
| [60,65) | 0.55 (0.50, 0.60) | 0.62 (0.56, 0.68) | 0.073 | 0.15 |
| [65,70) | 0.61 (0.55, 0.66) | 0.71 (0.65, 0.76) | 0.014 | 0.07 |
| [70,75) | 0.58 (0.51, 0.66) | 0.68 (0.61, 0.74) | 0.067 | 0.15 |
| [75,105) | 0.60 (0.52, 0.67) | 0.60 (0.52, 0.66) | 0.919 | 0.92 |

*‡*Generalized linear models; †Benjamini-Hochberg Procedure

**Table S5**. Predicted e-POSA proportions in males and females in each age group after adjusting for the effect of BMI.

| Age group | Female | Male | *P‡* | Adj. *P†* |
| --- | --- | --- | --- | --- |
| [2,5) | 0.19 (0.14, 0.26) | 0.20 (0.15, 0.25) | 0.86 | 0.86 |
| [5,10) | 0.31 (0.25, 0.37) | 0.26 (0.21, 0.32) | 0.19 | 0.66 |
| [10,15) | 0.33 (0.26, 0.41) | 0.36 (0.30, 0.42) | 0.54 | 0.73 |
| [15,20) | 0.35 (0.26, 0.47) | 0.42 (0.33, 0.51) | 0.33 | 0.66 |
| [20,25) | 0.30 (0.21, 0.41) | 0.24 (0.17, 0.32) | 0.34 | 0.49 |
| [25,30) | 0.30 (0.22, 0.38) | 0.24 (0.18, 0.32) | 0.34 | 0.49 |
| [30,35) | 0.30 (0.24, 0.37) | 0.26 (0.21, 0.32) | 0.38 | 0.49 |
| [35,40) | 0.31 (0.25, 0.36) | 0.25 (0.21, 0.30) | 0.10 | 0.49 |
| [40,45) | 0.29 (0.24, 0.33) | 0.23 (0.19, 0.27) | 0.07 | 0.49 |
| [45,50) | 0.24 (0.20, 0.28) | 0.23 (0.19, 0.27) | 0.66 | 0.72 |
| [50,55) | 0.22 (0.19, 0.26) | 0.22 (0.19, 0.26) | 0.94 | 0.94 |
| [55,60) | 0.24 (0.20, 0.27) | 0.20 (0.17, 0.24) | 0.17 | 0.49 |
| [60,65) | 0.15 (0.12, 0.18) | 0.18 (0.15, 0.22) | 0.15 | 0.49 |
| [65,70) | 0.22 (0.18, 0.26) | 0.25 (0.21, 0.29) | 0.39 | 0.49 |
| [70,75) | 0.21 (0.16, 0.26) | 0.24 (0.19, 0.29) | 0.41 | 0.49 |
| [75,105) | 0.22 (0.17, 0.27) | 0.19 (0.15, 0.23) | 0.33 | 0.49 |

*‡*Generalized linear models; †Benjamini-Hochberg Procedure

**Supplementary References**

1. American Academy of Sleep M. International Classification of Sleep Disorders. Diagnostic and Coding Manual. 2005:148-52.

2. Sateia MJ, Buysse DJ, Krystal AD, Neubauer DN, Heald JL. Clinical Practice Guideline for the Pharmacologic Treatment of Chronic Insomnia in Adults: An American Academy of Sleep Medicine Clinical Practice Guideline. J Clin Sleep Med. 2017;13(2):307-49. doi: 10.5664/jcsm.6470.

3. Berry RB, Budhiraja R, Gottlieb DJ, Gozal D, Iber C, Kapur VK, et al. Rules for scoring respiratory events in sleep: update of the 2007 AASM Manual for the Scoring of Sleep and Associated Events. Deliberations of the Sleep Apnea Definitions Task Force of the American Academy of Sleep Medicine. J Clin Sleep Med. 2012;8(5):597-619. doi: 10.5664/jcsm.2172.
